# Integrated miRNA, mRNA and protein expression analysis reveals the role of post-transcriptional regulation in controlling CHO cell growth rate

**DOI:** 10.1186/1471-2164-13-656

**Published:** 2012-11-21

**Authors:** Colin Clarke, Michael Henry, Padraig Doolan, Shane Kelly, Sinead Aherne, Noelia Sanchez, Paul Kelly, Paula Kinsella, Laura Breen, Stephen F Madden, Lin Zhang, Mark Leonard, Martin Clynes, Paula Meleady, Niall Barron

**Affiliations:** 1 National Institute for Cellular Biotechnology, Dublin City University, Dublin 9, Ireland; 2 Molecular Therapeutics for Cancer Ireland, Dublin City University, Dublin 9, Ireland; 3 Bioprocess R&D, Pfizer Inc., Andover, Massachusetts, USA

**Keywords:** Chinese hamster ovary, CHO cells, Growth rate, MicroRNA, mRNA, Microarray, Proteomics

## Abstract

**Background:**

To study the role of microRNA (miRNA) in the regulation of Chinese hamster ovary (CHO) cell growth, qPCR, microarray and quantitative LC-MS/MS analysis were utilised for simultaneous expression profiling of miRNA, mRNA and protein. The sample set under investigation consisted of clones with variable cellular growth rates derived from the same population. In addition to providing a systems level perspective on cell growth, the integration of multiple profiling datasets can facilitate the identification of non-seed miRNA targets, complement computational prediction tools and reduce false positive and false negative rates.

**Results:**

51 miRNAs were associated with increased growth rate (35 miRNAs upregulated and 16 miRNAs downregulated). Gene ontology (GO) analysis of genes (n=432) and proteins (n=285) found to be differentially expressed (DE) identified biological processes driving proliferation including mRNA processing and translation. To investigate the influence of miRNA on these processes we combined the proteomic and transcriptomic data into two groups. The first set contained candidates where evidence of translational repression was observed (n=158). The second group was a mixture of proteins and mRNAs where evidence of translational repression was less clear (n=515). The TargetScan algorithm was utilised to predict potential targets within these two groups for anti-correlated DE miRNAs.

**Conclusions:**

The evidence presented in this study indicates that biological processes such as mRNA processing and protein synthesis are correlated with growth rate in CHO cells. Through the integration of expression data from multiple levels of the biological system a number of proteins central to these processes including several hnRNPs and components of the ribosome were found to be post-transcriptionally regulated. We utilised the expression data in conjunction with *in*-*silico* tools to identify potential miRNA-mediated regulation of mRNA/proteins involved in CHO cell growth rate. These data have allowed us to prioritise candidates for cell engineering and/or biomarkers relevant to industrial cell culture. We also expect the knowledge gained from this study to be applicable to other fields investigating the role of miRNAs in mammalian cell growth.

## Background

Our understanding of the role microRNAs play in fundamental biological processes in both plants and animals has increased dramatically over the last decade [1]. Since the discovery of miRNA in *C*.*elegans*[2], the miRBase data repository has expanded to hold sequence data from over 21,000 mature miRNAs across 168 species [3]. These short, highly conserved RNA molecules (~22 nucleotides) form a layer of post-transcriptional control of gene expression, generally repressing translation [1] (via translational inhibition, transcriptional degradation and in some instances mRNA deadenylation [4]) or in rare cases actually enhancing translation [5]. Thus far, miRNAs have been implicated in a broad range of processes from cell cycle control [6] to apoptosis [7]. In addition, the effect of miRNAs on diseases such as cancer [8] and diabetes [9] has been intensively studied.

The complexity of miRNA target recognition remains a significant challenge to researchers. For instance, a single miRNA is estimated to target an average of 100–200 mRNAs [10] while a single mRNA transcript can be targeted by hundreds of miRNAs [11] and multiple miRNAs can cooperatively repress a range of targets [12]. The laborious experimental techniques required to confirm interaction between a miRNA and mRNA have necessitated the use of *insilico* target prediction to prioritise targets for wet-lab confirmation and to determine the potential function of a miRNA. The most widely applied algorithms including miRanda [13], Pictar [10] and TargetScan [14] use a combination of sequence complementarity of a transcript to a conserved region at the 5’ end of the miRNA spanning position 2 to position 7 (known as the “seed” region), thermodynamic feasibility of hybridisation and evolutionary conservation [1]. Until recently, active animal miRNA recognition sites were thought to be present solely within the 3’ UTR of an mRNA. However, recent evidence has suggested that sites also exist in the 5’ UTR [15], within the coding sequence [16], and in some cases these sites may be present in multiple locations within a transcript. Target prediction algorithms are undoubtedly valuable tools to researchers in the field providing a rapid appreciation of the potential processes impacted by a particular miRNA and prioritising potential direct targets for validation assays. Quantitative evaluation of algorithm performance has thus far proved difficult due to the limited number of experimentally confirmed targets. Previous studies have yielded less than encouraging false positive and false negative rates resulting from the use of algorithms [17,18] prompting an increasing focus on combining multiple expression profiling datasets with *insilico* target prediction [19].

The Chinese hamster ovary (CHO) cell has been utilised extensively for the last 20 years in the biopharmaceutical industry and has become the cell type of choice for production of recombinant proteins for medical applications due to safety of use, rapid growth characteristics and the ability to secrete large quantities of correctly folded proteins. The majority of industrial advances have thus far stemmed from improvements in cell culture media, vectors and the design of bioreactors [20]. Several research groups have in recent years been interested in manipulating CHO at the molecular level to improve protein production efficiency and create diagnostic tools to monitor manufacturing processes. The recent publication of the CHO genome [21] along with sequence from similar initiatives [22,23] promises to facilitate analyses at all levels of the CHO biological system not only through direct analysis of genomic sequence but also through the improvement of analytical platforms such as microarrays and mass spectrometry.

Since the first report of altered miRNA expression in CHO as a result of modifying bioprocess conditions [24], the number of publications in this area has increased steadily. The attraction of miRNA based cell engineering arises from the potential of miRNAs to alter an entire pathway or indeed pathways, to enhance industrially beneficial phenotypes. Various studies have focussed on miRNA sequence analysis, determining homology to other species and location of genomic loci via next generation sequencing technologies [25-27]. To date, miRNAs have been associated with several important bioproduction phenotypes including growth rate [28], productivity [29] and apoptosis [30]. In this study, we elucidate new and expand existing knowledge on the contribution of miRNA-mediated regulation to CHO cellular growth rate. Our previous work has shown that building CHO cell density to a high level in the bioreactor is intimately linked to the final volumetric titre of product and can in some cases be more important than the intrinsic productivity rate [31].

The experimental design and stringently controlled panel of samples used here ensures that the data presented in this study are applicable to the investigation of mammalian cell growth beyond the bioprocessing field. Firstly, the cells under investigation were selected from the same clonal population following a process of repeated passaging and monitoring of growth rate in order to minimise non-growth related variation. A set of these sister clones spanning a continuous range of low to high growth rates was selected for analysis. The second crucial aspect of our approach was to measure expression of the transcriptome (mRNA & miRNA) and proteome in parallel. While the number of studies combining miRNA, protein and mRNA expression data are limited in comparison to those comprising of two data types (i.e. miRNA & mRNA or miRNA & protein), it is evident that the use of all of 3 of these data types can facilitate the identification of direct miRNA targets [32] and enhance our understanding of the biological role of miRNAs [33]. Similar to those studies, we analyse the expression data in conjunction with miRNA target prediction algorithms in order to decrease false positives, reduce the effects of experimental noise and ultimately to increase the likelihood of finding direct miRNA targets.

It is intended that this work will contribute to the understanding of those biological processes driving mammalian cell growth as well as supporting other researchers in the selection of candidates for miRNA-target confirmation assays.

## Results

The growth rates of the final sample set spanned a range of 0.011 to 0.044 hr^-1^ with mean productivity = 24 (± 3) pg protein/cell/day. Consistent behaviour in the samples subjected to expression profiling in terms of growth rate and productivity was ensured by monitoring over 40 passages. By choosing sister clones derived from the same transfection pool with similar recombinant protein production rates and differing only in growth rate we sought to eliminate noise and expose those variations related to the proliferation phenotype. To prioritise miRNAs, mRNAs and proteins associated with cell growth, we separated the dataset into 15 “fast” (≥ 0.025 h^-1^) and 15 “slow” (≤ 0.023 h^-1^) samples. Note: 3 outlying “slow” samples (biological replicates) within the LC-MS/MS dataset were removed following quality control using principal components analysis (data not shown). To equalise the sample numbers on both sides of the proteomic differential expression analysis a single fast growing clone (3 biological replicates) was selected at random and removed leaving 12 fast versus 12 slow samples (Additional file 1).

**Figure 1:**
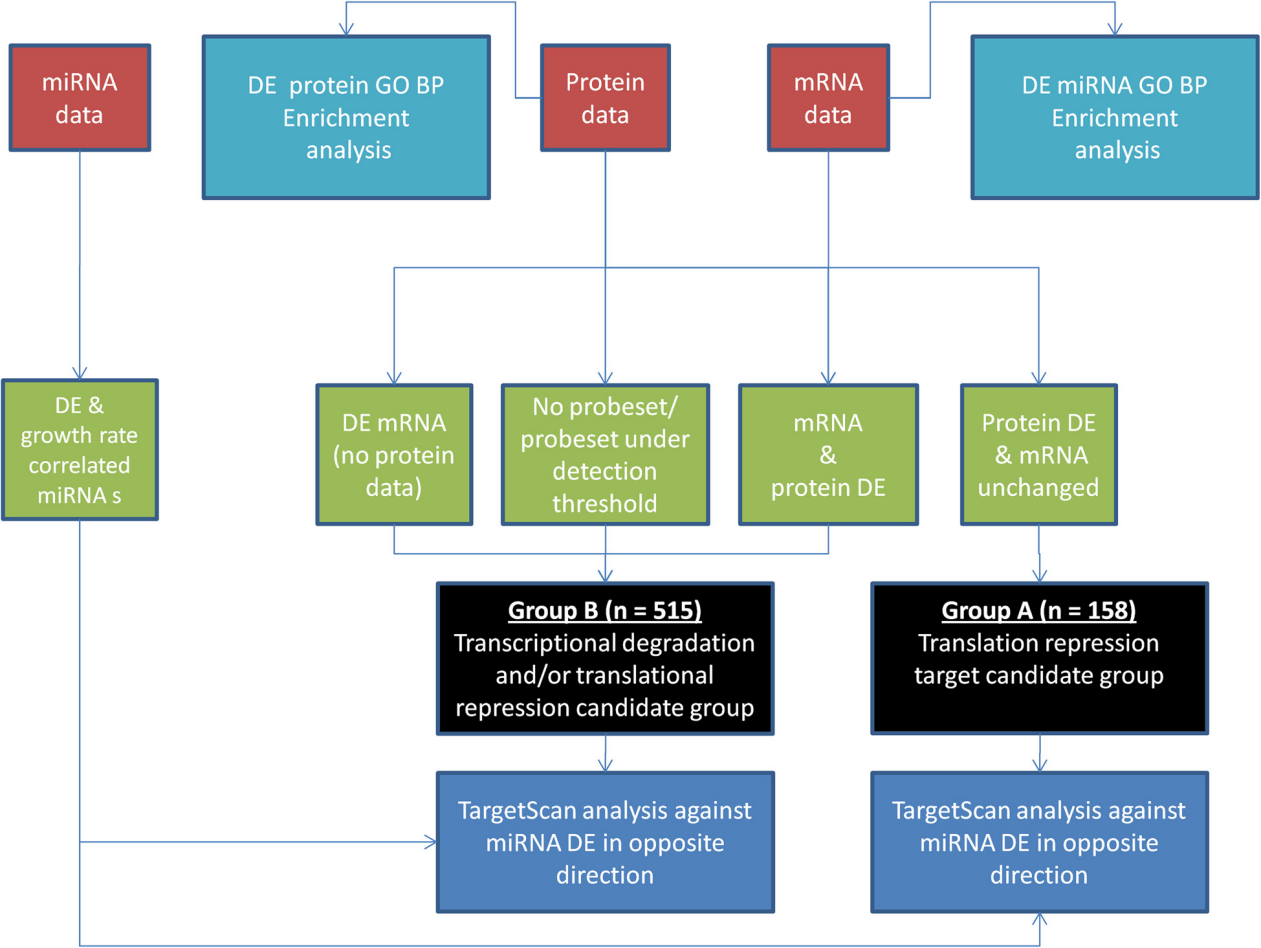
**Data analysis overview.** Stage 1: Differential expression analysis between “fast” and “slow” groups for each of the 3 datasets. Stage 2: Enrichment analysis using DAVID to determine overrepresented GO biological processes in the DE mRNA and protein lists. Stage 3: mapping of DE proteins to microarray probesets, output of two target groups; Group A contains potential targets of miRNA translational repression (protein DE without mRNA change), Group B contains translation repression and/or transcriptional degradation targets (contains DE mRNA & DE proteins where no microarray information was available/probeset was below detection level or both mRNA and protein were DE). Stage 4: TargetScan analysis of both groups against miRNAs DE in the opposite direction.

Figure 1 shows an overview of the data analysis strategy used in this study. We began by prioritising candidates with respect to growth rate from the miRNA, mRNA and proteomic datasets in isolation. Enrichment analysis against GO was conducted using the DAVID interface for the resulting DE mRNA and protein lists to determine if any biological processes were overrepresented. The availability of both mRNA and protein data acquired in parallel is particularly advantageous in identifying targets undergoing potential miRNA translational repression.

Prior to target prediction against the DE miRNA list, we separated protein and mRNA targets into two groups. “Group A” contains those targets where a degree of post-transcriptional regulation was observed (possibly via miRNA mediated translational repression). The proteins in Group A were DE between the fast and slow clones, their respective mRNAs were expressed above the microarray detection threshold but no change in mRNA expression was observed. The second group of candidates, referred to as “Group B” were comprised of (1) DE proteins where the probeset was under the detection threshold or not present on the chip, (2) DE mRNAs where the corresponding protein was not identified within the fraction analysed by LC-MS/MS and (3) candidates where differential expression was observed at both the mRNA and protein level (Figure 1). It is therefore likely that Group B contains a mixture of targets that could be undergoing miRNA translational repression, transcript destabilisation or indeed alteration due to non-miRNA-mediated processes. Group B candidates were considered of lower priority than Group A not because we expected a lower proportion of predicted miRNA targets, but because of incomplete evidence at the protein and mRNA levels or both the protein and mRNA were DE. The final stage in analysis involved *insilico* target prediction with TargetScan 6.1 [14] for Groups A and B. Each protein/mRNA from the two groupings was compared with the predicted targets of anti-correlated DE miRNAs.

### miRNA expression levels are associated with variations in the rate of CHO cell growth

**Figure 2:**
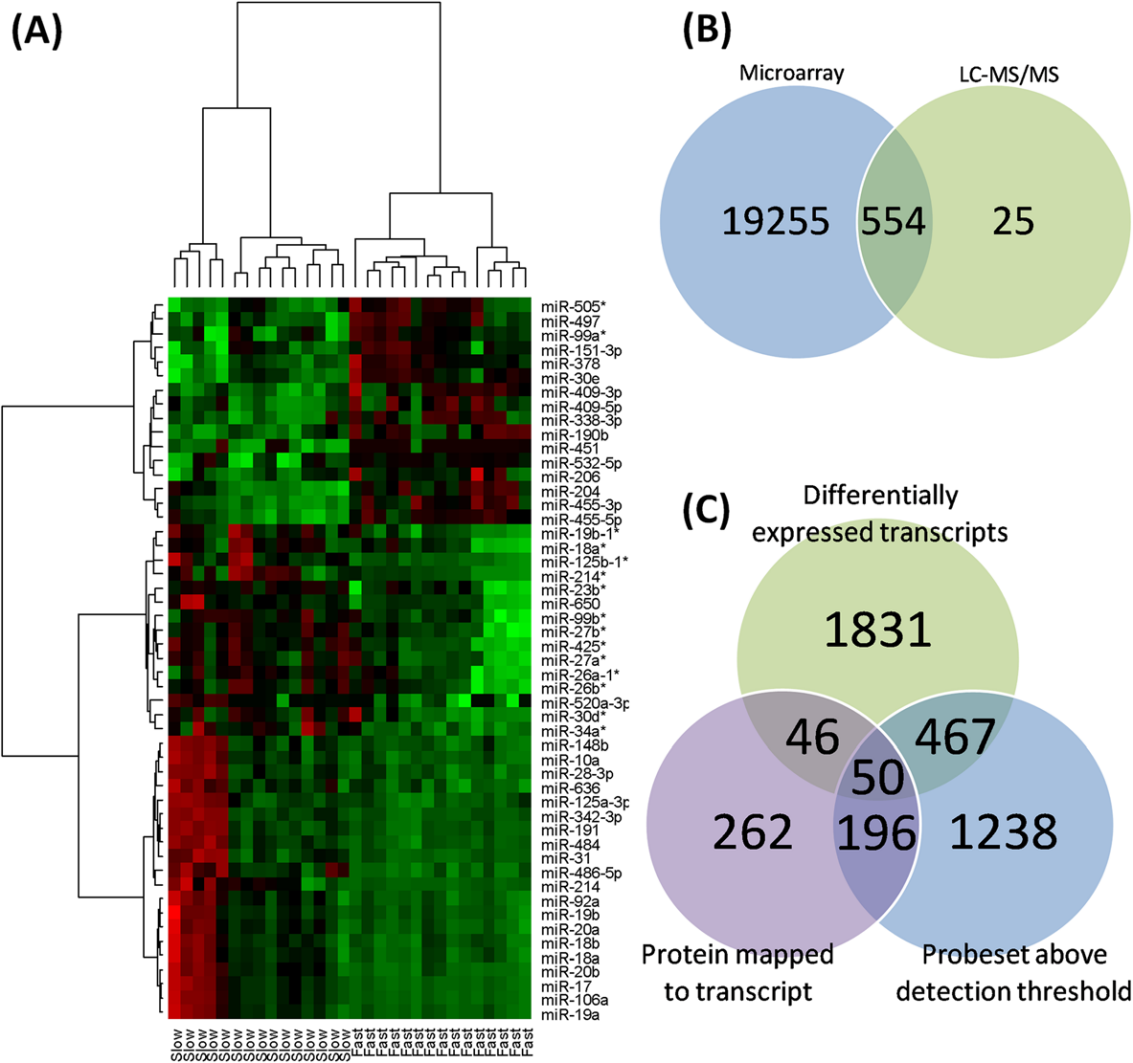
**miRNA DE analysis heatmap and mRNA**-**proteomic mapping.** miRNA expression profiling identified 51 miRNAs that were DE and correlated to sample growth rate. (**A**) HCA analysis confirmed that those DE and correlated genes separate the samples into fast and slow groups (red indicates diminished miRNA expression and green indicates increased miRNA expression). (**B**) 260 DE proteins mapped to one or more probesets. (**C**) 196 probesets corresponding to 158 DE proteins expressed above the detection threshold and unchanged at the mRNA level between the fast and slow groups.

The expression of 667 miRNAs was measured across a group of CHO cell clones with varying rates of cell growth. Differential expression analysis revealed 93 miRNAs (76 upregulated & 17 downregulated) that exhibited statistically significant alternations in expression between the fast and slow growing groups. We further prioritised these DE miRNAs through calculation of the Pearson correlation coefficient (PCC) between ΔC_T_ values and the sample growth rate. Only those miRNAs with a PCC ≤ −0.4 or ≥ +0.4 were retained for further analysis (Additional file 2). In total, we identified a high priority set of 51 miRNAs that were both DE between fast and slow growing CHO cells and exhibited a degree of correlation with growth rate across the set of clones. The expression of 16 of these miRNAs diminished when the growth rate increased, while the expression of 35 miRNAs increased as growth rate increased (Figure 2A).

Of those miRNAs found to be DE in this study, a number have been associated with cellular growth previously [34-40]. Several of these upregulated miRNAs form part of the miR-17-92 cluster (e.g. miR17, miR20a, miR20b, miR18a, miR18b and miR106a), a well studied group of miRNAs linked to cancer [41]. It has been shown that c-myc [42] and N-myc [43] directly activate the mir-17-92 cluster, while in contrast repression of the cluster by p53 has been demonstrated [39]. CHO is a highly proliferative cell line in general having been cultured in vitro for over 50 years [44] and previous studies have shown that the miR-17-92 cluster is highly expressed [26]. The data presented in this study indicates a relationship between increased miR-17-92 cluster member expression levels and rapidly proliferating CHO cells. Several miRNAs, including miR-204, miR-338-3p, miR-497, miR-30e and miR-206 are downregulated as growth rate increases in agreement with independent studies in other systems [38,45-47]. miR-451 (downregulated at higher growth rates in this study) has been shown to inhibit growth and induce apoptosis [37,48]. Godlewski et al. demonstrated that in glioma cells miR-451 expression is correlated to glucose levels and upon glucose depletion a downregulation of miR-451 is observed along with slower proliferation and increased survival [35]. In contrast, downregulation of miR-451 in CHO is observed here at higher growth rates, exemplifying the previously recognised cell-specific nature of miRNA expression.

### Proteomic and transcriptomic profiling reveal several overrepresented biological processes related to mRNA processing and translation

285 non-redundant proteins (180 upregulated and 105 downregulated) were found to be DE between fast and slow growing CHO cell clones (Additional file 3). Following differential expression analysis of the mRNA data we identified 432 DE non-redundant annotated transcripts (229 upregulated and 203 downregulated) (Additional file 4). 44 proteins were also dysregulated at the mRNA level (29 upregulated and 15 down regulated, corresponding to 50 DE probesets (Figure 2C)). Correlation between both datasets was observed for 11 of the 44 proteins in this group (9 upregulated and 2 downregulated).

GO biological processes found to be overrepresented within the DE protein list included translation, ribosomal biogenesis and energy metabolism (Table 1 and Additional file 5). Analysis of DE mRNAs revealed similar categories to that of the protein list (e.g. RNA processing and RNA splicing) and also the enrichment of mitotic cell cycle genes (Table 2 and Additional file 5). The presence of GO biological processes including translation and mRNA processing highlighted here agree with reports that the availability of cellular translation machinery to process mRNA and produce the proteins required to generate new biomass is at least partly responsible for variation in cellular growth rate [49,50]. For instance, the upregulation of numerous ribosomal proteins (RP) was observed (e.g. RPL14, RPS15 and RPL15). Several previous studies have demonstrated the central role of RPs in processes beyond translation [51] including the regulation of cell growth and division rates [52]. RP expression has also been shown to be modulated by oncogenes (e.g. c-Myc [53]) and tumour suppressor proteins (e.g. p53 [54]). The translation of these proteins and their role in ribosome biogenesis is known to be controlled through a variety of post-transcriptional and post-translational factors [55].

**Table 1:** Protein enrichment analysis

**GO ID**	**GO Term**	**P**-**value**	**BH adj**.
0006414	translational elongation	7.57×10^-42^	1.21×10^-38^
0006412	translation	1.58×10^-22^	1.26×10^-19^
0006091	generation of precursor metabolites and energy	4.85×10^-16^	2.36×10^-13^
0055114	oxidation reduction	1.29×10^-15^	5.31×10^-13^
0009060	aerobic respiration	7.32×10^-13^	2.33×10^-10^
0006396	RNA processing	3.20×10^-11^	8.51×10^-09^
0006732	coenzyme metabolic process	5.77×10^-11^	1.31×10^-08^
0045454	cell redox homeostasis	1.04×10^-10^	2.07×10^-08^
0008380	RNA splicing	1.04×10^-10^	1.85×10^-08^
0045333	cellular respiration	2.80×10^-10^	4.46×10^-08^

Top 10 most significantly enriched GO categories for DE proteins. See Additional file 5 for complete DAVID output.

**Table 2:** mRNA enrichment analysis

**GO ID**	**GO Term**	**P**-**value**	**BH adj**.
0000278	mitotic cell cycle	2.38×10^-05^	0.020
0006396	RNA processing	5.18×10^-05^	0.022
0016071	mRNA metabolic process	4.03×10^-05^	0.023
0008380	RNA splicing	9.95×10^-05^	0.028
0034621	cellular macromolecular complex subunit organization	8.58×10^-05^	0.029
0006397	mRNA processing	1.79×10^-05^	0.030

Enriched GO categories for DE mRNAs. See Additional file 5 for complete DAVID output.

mRNA processing and splicing processes were also found overrepresented within both the protein and transcript differential expression lists. Alternative splicing is understood to represent an important stage at which regulation of translation can be mediated. Furthermore, RNA splicing is now recognised as a central step in gene expression whereby virtually all precursor mRNAs (pre-mRNAs) undergo alternative splicing, resulting in a complex level of expression regulation [56]. Specific splicing factors are known to be important for cell cycle control, for instance multiple splice variants of the p53 tumour suppressor are DE in cancer [57]. We observed the post-transcriptional upregulation of YBX1/YB1 in rapidly growing CHO cells; this transcription factor is associated with cancer susceptibility [58] and known to play a role in splice site selection [59]. Previous studies reporting an increase or decrease in tumour cell growth upon over or under-expression of this protein respectively [60] are in-agreement with the evidence reported here.

In recent years, various studies have demonstrated the role of RNA-binding proteins (RBPs) in the regulation of groups of transcripts by shuttling them efficiently through processes such as mRNA splicing, transport and ultimately translation [61]. This post-transcriptional regulation of multiple mRNAs, termed “RNA regulons”, is thought to allow the cell to respond rapidly to environmental changes. One class of RBPs, known as hnRNPs, interact with large numbers of pre-mRNAs to form hnRNP-RNA complexes containing combinations of at least 20 hnRNPs. Further examination of those RNA processing and splicing GO categories found to be enriched in this study revealed the presence of multiple hnRNPs DE at both the protein (e.g. hnRNPM, hnRNPC, hnRNPAB, and hnRNPK) and mRNA (e.g. hnRNPD and hnRNPR) level (Additional files 3 and 4).

### Separation of DE mRNA and DE proteins into target Groups A and B

To compare fluctuations in protein abundance with transcript expression we identified probesets on the array representing the DE proteins identified by LC-MS/MS. In the case of multiple probesets targeting an individual protein, the probeset with the highest expression was selected to represent that protein. 554 probesets corresponding to 260 proteins were identified (probesets for 25 proteins were absent from the microarray) (Figure 2B). Group A contained 196 probesets corresponding to 158 unique DE proteins (Figure 2C) where a degree of post-transcriptional regulation was observed. Group B was comprised of 515 candidates including DE mRNAs, DE proteins where no probeset was available or the mRNA was undetected and candidates DE at both the mRNA and protein level. For the 44 Group B targets where both the mRNA and protein was DE only the protein data (i.e. direction) was utilised for miRNA target overlap.

### *In*-*silico* analysis identifies a number of transcriptomic and proteomic targets of priority miRNAs

**Figure 3:**
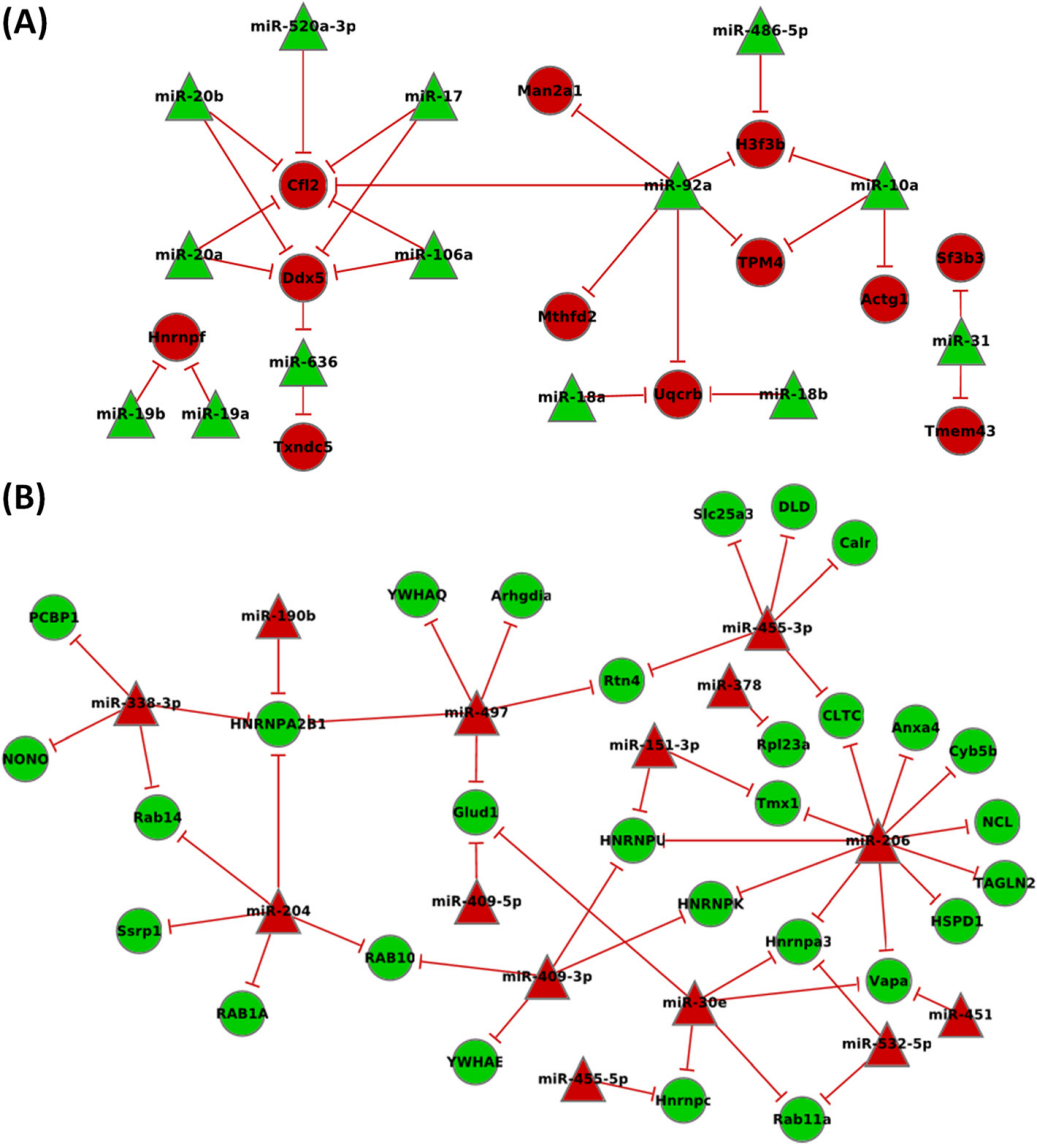
**Summary of TargetScan predicted Group A miRNA targets.** TargetScan prediction analysis for (**A**) upregulated (green) and (**B**) down (red) miRNAs against post-transcriptionally regulated proteins. The prioritisation of these targets was possible only through integration of the mRNA and protein datasets.

TargetScan was used to determine if any of the proteins or mRNAs present within Group A (n = 158) or Group B (n = 515) were predicted to be targeted by anti-correlated miRNAs. In total, 22 upregulated and 14 downregulated priority miRNAs were present within the TargetScan database and utilised for prediction. 41 out of 158 of the Group A proteins (25.9%) were predicted to be direct targets of one or more anti-correlated miRNAs (Additional file 6). A network overview of the TargetScan predictions for Group A is shown in Figure 3 to summarise the potential interactions. Each protein is represented by a circular node and each miRNA by a triangular node; a line connects those proteins and miRNAs predicted to have an interaction. Target prediction was carried out for both the upregulated miRNAs vs. downregulated proteins (Figure 3A) and downregulated miRNAs vs. upregulated proteins (Figure 3B). For Group B targets 133 of the 515 (25.8%) mRNA/proteins were predicted to be targeted by one or more oppositely correlated miRNAs (Additional file 7).

We would not expect all candidates to be predicted targets of miRNAs for a number of reasons. It is likely differential expression of mRNA and proteins is occurring due to a variety of non-miRNA related processes. Furthermore the dysregulation of proteins such as hnRNPs suggests the likelihood of non-miRNA post-transcriptional regulation. The design of the TargetScan algorithm, considered to be amongst the most stringent methods, limits the numbers of in-silico predicted targets. The 15 “star” miRNAs found to be dysregulated and correlated to growth rate in this study could not be predicted as the TargetScan database does not contain entries for these miRNAs. In addition the algorithm only considers target sites within the 3’ UTR and therefore excludes potential target sites within the coding region or at the 5’ end of a transcript [14].

**Figure 4:**
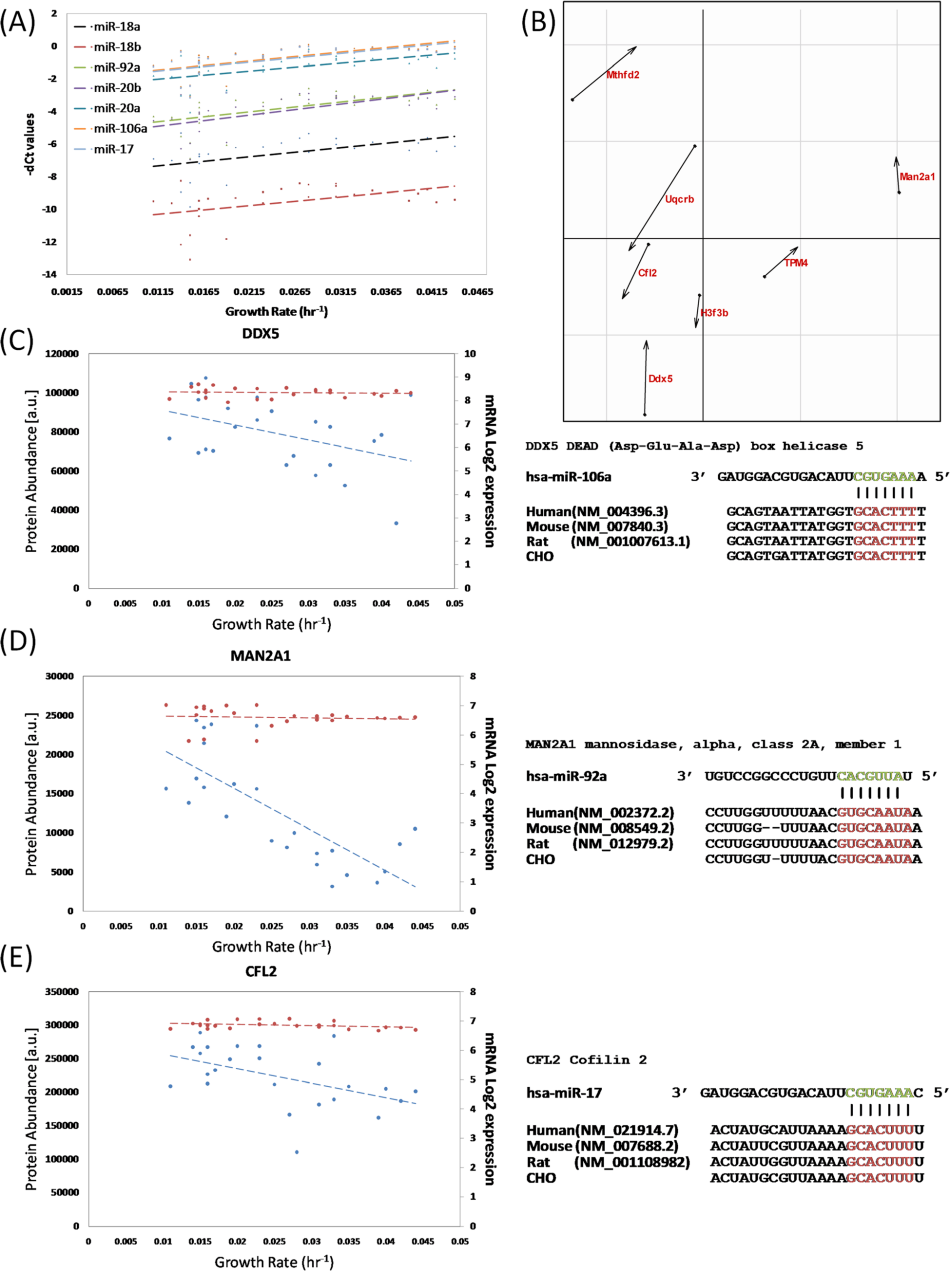
**Group A proteins downregulated and predicted to be targeted by miR17**-**92 cluster members.** (**A**) miR-17-92 cluster expression increases as growth rate increases. (**B**) Normalised CIA score plot showing the divergence between mRNA and protein expression profiles for miR17-92 TargetScan predictions. Each predicted target is represented by an arrow, the length of which corresponds to the divergence between mRNA (circular base) and protein (arrowhead) expression across the dataset. Potential miRNA 17–92 cluster mediated post-transcriptional repression of (**C**) DDX5, (**D**) MAN2A1 and (**E**) CFL2. For each of the three targets the mRNA expression (red) remains constant while the protein expression decreases (blue) for the 24 samples were both mRNA and protein data was available. The conserved (human, mouse, rat and CHO) binding site to the seed region of the miRNA cluster is shown to the right for each protein.

**Figure 5:**
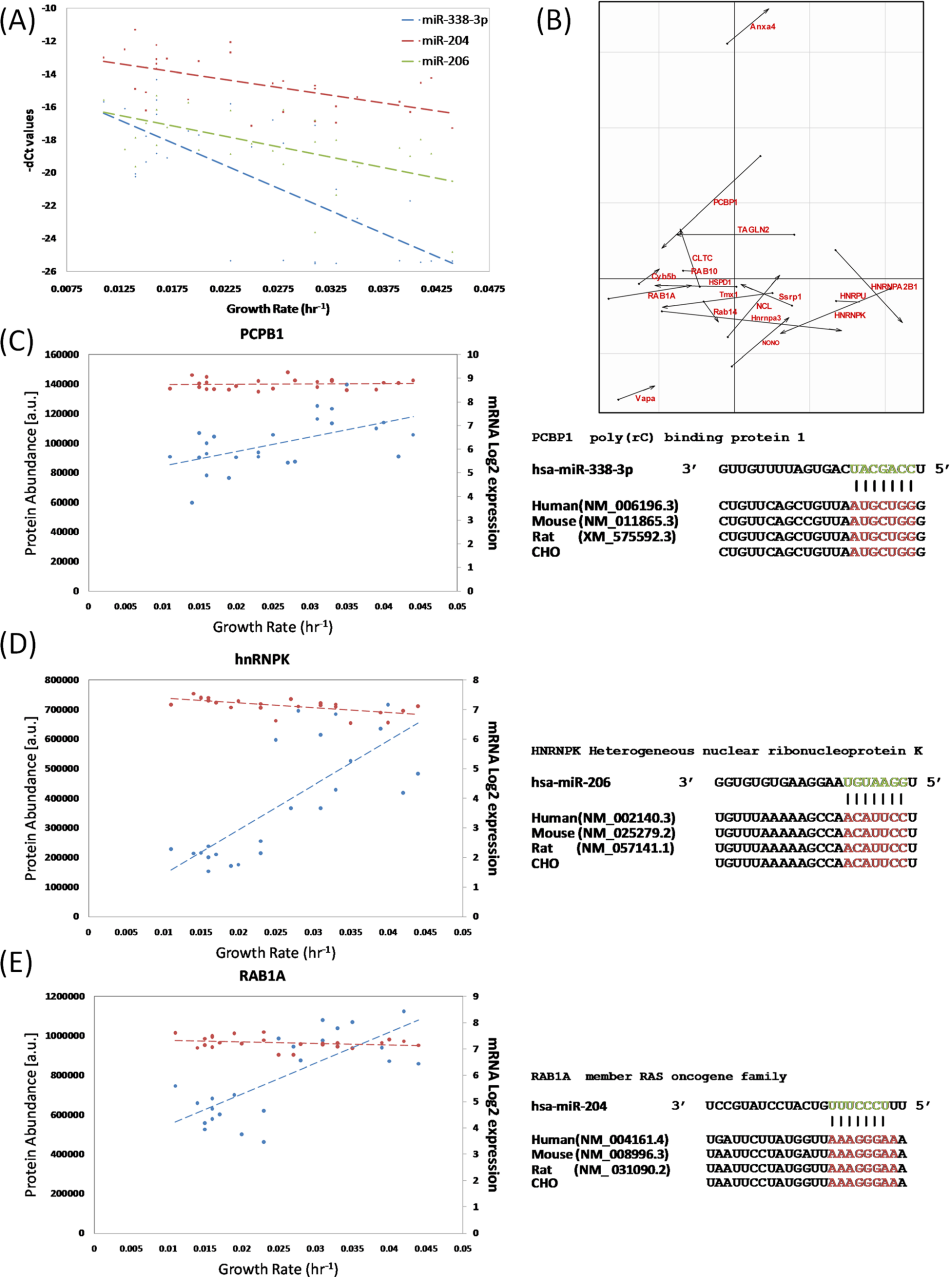
**Group A proteins upregulated and predicted to be targeted by selected downregulated miRNAs.** (**A**) The expression of miR-338-3p, miR-206 and miR-204 decreases as growth rate increases. (**B**) Normalised CIA score plot showing the divergence between mRNA and protein expression profiles for miR-338-3p, miR-206 and miR-204 TargetScan predictions. Each predicted target is represented by an arrow, the length of which corresponds to the divergence between mRNA (circular base) and protein (arrowhead) expression across the dataset. Potential post-transcriptional regulation of (**C**) PCBP1, (**D**) hnRNPK and (**E**) RAB1A. For each of the three targets the mRNA expression (red) remains constant while the protein expression increases (blue) for the 24 samples where both mRNA and protein data was available. The conserved (human, mouse, rat and CHO) binding site to the seed region of the miRNA cluster is shown to the right for each protein.

The expression of several members of the miR-17-92 cluster was found to increase as growth rate increased (Figure 4A). Coinertia analysis (CIA) was carried out on the 7 TargetScan predicted targets anti-correlated with miR-17-92 expression to demonstrate the disparity between mRNA and protein expression across the dataset without applying subjective DE criteria (Figure 4B). The two input (protein & mRNA) data matrices for CIA were comprised of 24 samples for 7 predicted miRNA targets. The expression profiles of three of these targets at the mRNA and protein level are shown: DDX5 (Figure 4C), MAN2A1 (Figure 4D) and CFL2 (Figure 4E). The mRNA expression of each of the three genes remains constant as growth rate increases, while protein expression decreases. The corresponding 3’ UTR transcript sequence alignment with predicted miRNA seed region and conservation across human, mouse, rat and CHO is also shown for each of the three proteins. We also examined the expression profiles and sequences for those proteins predicted to be targeted by three miRNAs found to be downregulated as growth increases, miR-338-3p, miR-204 and miR-206 (Figure 5A). TargetScan predicted 18 proteins to be directly targeted by the 3 miRNAs, therefore the CIA input data consisted of two matrices corresponding to 18 mRNAs/proteins across the 24 samples (Figure 5B). Two hnRNP proteins identified within enriched categories from the GO analysis were examined in detail. Poly(rC)-binding protein 1 (PCBP1), also known as hnRNPE1, is a predicted target of miR-338-3p. As can be seen, protein abundance increases as growth rate increases while the mRNA levels remain unchanged (Figure 5C). The TargetScan predicted conserved binding site is shown for the predicted miR-338-3p PCBP1 interaction. hnRNPK was also found to exhibit post transcriptional regulation; once again the mRNA expression remains constant while protein expression increases (Figure 5D). TargetScan identified conserved miR-206 and miR-409-3p (data not shown) binding sites in the hnRNPK 3’ UTR, both of these miRNAs are known to suppress cellular proliferation [40,62,63]. These results correlate well with a previous study demonstrating that downregulation of hnRNPK decreases cellular proliferation [64]. Subtle interplay between hnRNPs has been shown to play a role in miRNA biogenesis [65,66] and miRNAs can act as “decoys” to disrupt hnRNP-mediated translation inhibition [67]. The results obtained here suggest that miRNAs may also directly regulate the translation of hnRNP proteins during cellular growth. The mRNA and protein expression profile of RAB1A and sequence alignment of the conserved seed region match with miR-204 is shown below (Figure 5E). Numerous Ras associated binding (Rab) - GTPases were also found to be DE at the protein (RAB6A, RAB5B, RAB35, RAB2A, RAB1A, RAB14, RAB11A and RAB10) and mRNA level (RAB18). These proteins play an important role in processes such as vesicle transport, signal transduction and cytoskeleton formation [68]. Several of these Rab (RAB14, RAB1A, RAB10, RAB11A) proteins were post-transcriptionally regulated and were predicted to be targeted by miRNAs downregulated at higher growth rates (miR-204, miR-338-3p, miR-409-3p, and miR-30e).

## Discussion

To investigate the impact of miRNA, mRNA and protein expression on cellular growth rate we have employed an integrative methodology to combine data from several global profiling technologies with bioinformatics analysis. We sought to minimise variation through the use of carefully selected Mab-secreting CHO cell lines that, in spite of spanning a wide range of growth rates, were derived from the same transfection pool. Furthermore the abundance of miRNA, mRNA and protein was determined in parallel on identical samples to further reduce biological noise. The experimental approach may be applicable to the study of cell growth in other eukaryotic systems and prove useful in elucidating mechanisms of cellular proliferation in general.

The most crucial aspect of the experimental design is the combination of data from multiple expression profiling methods, genomic sequence and *insilico* prediction to study miRNA function. Both proteomic mass spectrometry and mRNA microarrays have been used previously to study miRNA function, however both methods when used in isolation, suffer from several disadvantages as noted by previous researchers in the area [32,69]. For instance quantitative mass spectrometry based proteomics yields in the order of hundreds of DE proteins and depending on the prefractionation method may not detect many of the low abundant proteins or integral membrane proteins. On the other hand gene expression analysis using microarrays provides a wide coverage of mRNAs but post-transcriptional processes may be missed. In terms of the study of the role of miRNAs both methods when used in isolation rely heavily on computational methods to predict miRNA interaction and prioritise potential direct targets. The availability of data on multiple levels of the biological system allows us to identify targets that would not have been identified by a single dataset. Moreover, prioritisation of potential targets undergoing classical miRNA-mediated translation repression can only be achieved through the integration of both the mRNA and protein datasets.

The availability of a combined profiling approach could reduce the false negative or false positive rates associated with *insilico* prediction as well as enriching priority candidate cohorts for functional validation. For example, a recent study demonstrated the enhancement of ribosomal subunit translation and ribosome biogenesis upon miR-10a binding via a “non-seed” site at the transcript 5’ UTR [5] and confirmed that ribosome formation can be modulated by miRNA to some extent. In this study miR-10a was both upregulated (FC = 2.12) and positively correlated (PCC = 0.53) with cellular growth rate, and several ribosomal proteins previously identified as mir-10a targets showed some evidence of post-transcriptional regulation. We found no predictive evidence of miR-10a interaction with those RPs using TargetScan as the algorithm searches for the presence of sites conforming to classical miRNA seed based rules within 3’ UTR. In addition, RP 3’ UTRs tend to be relatively short.

Rab14 is an experimentally validated direct target of miR-451 and has been shown to activate tumour suppression [37]. miR451 was downregulated and the Rab14 protein upregulated (while mRNA expression remained constant) in fast growing CHO cells. The Rab14-miR451 3’ UTR binding site is poorly conserved and therefore not ranked highly by TargetScan; in cases such as this the availability of evidence from multiple profiling datasets could be used to prioritise poorly conserved miRNA interactions.

## Conclusions

In summary, we have analysed a range of production CHO cell clones to investigate the role of miRNAs in growth rate variation. The antibody producing clones under analysis were chosen to control for several possible confounding factors. We employed global miRNA, mRNA and proteomic expression profiling in parallel in order to integrate three levels of the biological system. Analysis of the datasets in isolation provided information on the processes involved in cell growth which included ribosome biogenesis, translation and mRNA processing, which were to some degree regulated post-transcriptionally. The ability to compare mRNA and proteins in identical samples with respect to miRNA allowed us to identify potential miRNA targets, and highlight translational repression targets which could not have been identified using a single dataset. Moreover, the use of multiple profiling datasets could permit the identification of non-seed miRNA targets complementing computational prediction tools and reducing the false positive and false negative rates. While this study is particularly relevant for the bio-pharmaceutical industry by prioritising a number of potential miRNA cell engineering candidates, the experimental design ensures that the data generated and knowledge gained on biological processes driving cellular growth are applicable to other mammalian systems.

## Methods

### Cell culture

Clonal cell lines were grown in batch shake flask suspension culture (60 ml working volume) in proprietary serum-free media at 37°C, without feeds or temperature shift. Each clone was grown in triplicate flasks and samples collected at a single time point (72 hrs - mid/late log). Cell counts and viability were determined using trypan blue exclusion and a hemocytometer. Growth rates (in reciprocal hours; h^-1^) were calculated according to the following equation:

(1) $$growth\;rate=\left(\frac{ln\left(density\;2\right)-ln\left(density1\right)}{time\;2-time\;1}\right)$$

In total, 30 clones were selected for expression profiling ranging from 0.011 to 0.044 hr^-1^. 15 clones were designated as “fast” (≥ 0.025 h^-1^) and 15 clones designated as “slow” (≤ 0.023 h^-1^) (Additional file 1).

### qPCR screening of miRNA expression

Total RNA was extracted from 10^6^ cells using Trireagent^TM^ (Sigma-Aldrich), resuspended in nuclease free water, quantified on a Nanodrop^TM^ spectrophotometer and checked for integrity on a Eukaryote Total RNA Nano Bioanalyzer chip (Agilent). TaqMan Low-Density Array cards (TLDAs) (Human MicroRNA A&B Cards V2.0) were run as per the manufacturer’s guidelines (Applied BioSystems). Each card consists of 384-wells containing primers designed against individual miRNAs. 100 ng of total RNA was reverse transcribed in 2 individual multiplex reactions. These cDNA mixes were subjected to 12 cycles of pre-amplification with pre-amp primer pools and then used to load the TLDA card. PCR was performed on an AB7900 real time instrument for 10 min at 95°C followed by 40 cycles of 30 sec at 97°C and 1 min at 60°C.

The R statistical software environment (http://www.r-project.org) and the HTqPCR bioconductor package were utilised for data analysis [70]. Mamm-U6 expression was used to normalize across the samples and differential expression was calculated using the 2^-ΔΔCt^ method. miRNAs with a fold change ≥ 1.2 in either direction between the fast and slow groups with a Benjamini-Hochberg (BH) adjusted p-value of < 0.05 were considered significant. The Pearson correlation coefficient (PCC) between miRNA ΔC_T_ and sample growth rate (Additional file 1) was also utilised as an additional filter.

### Microarray analysis

Gene expression analysis was carried out on the proprietary CHO-specific WyeHamster3a oligonucleotide microarray. The array contains 19,809 probesets corresponding to 132 control sequences, 11,857 probesets annotated to mouse, rat and/or human Unigene IDs (9,098 non-redundant genes) and 7,820 unannotated probesets. The methodology and criteria used for total RNA purification, cRNA sample processing and hybridisation to hamster microarrays have been previously described [71]. All microarray data were pre-processed as described previously [31]. Prior to data-analysis probesets that did not reach the detection threshold (fluorescence level ≥ log2 (100) for at least 1 sample) were identified and designated undetected. The remaining probesets were considered differentially expressed between the fast and slow groups if a fold change ≥ 1.2 in either direction along with a BH adjusted p-value < 0.05 was observed. The microarray data used in this study have been deposited in the NCBI GEO database (GSE37251).

### Proteomics sample preparation, LC-MS/MS and data analysis

Sample preparation and label-free LC-MS was carried out as previously described [72]. Data analysis was performed using Progenesis label-free LC-MS software version 3.1 (NonLinear Dynamics LTD, Newcastle upon Tyne, UK) as recommended by the manufacturer (see http://www.nonlinear.com for further background information regarding alignment, normalisation, calculation of peptide abundance, etc.). Briefly, the raw MS data is processed as follows; a run is selected that is representative of the data, to which the LC retention times of all the other samples within the experiment are aligned. The Progenesis peptide quantification algorithm calculates normalised peptide abundances as the sum of the peak areas within each peptide isotope boundary. Protein abundance is calculated from the sum of all unique peptide abundances for an individual protein on each run.

A number of criteria were used to filter the data before exporting the MS/MS output files to MASCOT (http://www.matriscience.com) for protein identification; only peptide features with a p-value < 0.05 (determined via an ANOVA) between experimental groups, mass peaks (features) with charge states from +1 to +3, and greater than 3 isotopes per peptide were retained. All MS/MS spectra were exported from Progenesis software as a MASCOT generic file (.mgf) for peptide identification with MASCOT (version 2.2) and searched against the BB-CHO specific database [73]. The search parameters used were as follows: peptide mass tolerance set to 20 ppm, MS/MS mass tolerance set at 0.5 Da; up to two missed cleavages were allowed, carbamidomethylation set as a fixed modification and methionine oxidation set as a variable modification. Peptides with ion scores of 30 and above were re-imported into the Progenesis LC–MS software for further analysis. Only proteins with ≥ 2 peptides matched, a ≥ 1.2 fold difference in abundance in both directions and a p-value < 0.05 were considered to be DE.

### Coinertia analysis

Coinertia analysis is a multivariate statistical method utilised to compare datasets with different measurement sources on the same objects/samples. There have been several examples of CIA applied to “omics” data to date including a comparison of transcriptomic and proteomic data [74] and prediction of miRNA interactions from gene expression analysis [75]. Briefly, CIA attempts to locate the axes of maximal co-variance between the proteomics and transcriptomic data from parallel samples. CIA was carried out using the MADE4 R package [76]. Gene expression and proteomic data were log2 scaled and mean centred prior to analysis. In this study we employ CIA to visualise the disparity between transcript and protein abundance across the dataset in an unsupervised manner negating the requirement for arbitrary thresholds. The input for the CIA analysis was two matrices equal to n × p, where n = sample number and p = number proteins/mRNA potentially targeted by the miRNAs of interest. Note: Only 24 matched mRNA protein samples were used for CIA due to the presence of outlying samples in the LC-MS/MS dataset. Following CIA the normalised scores were plotted, each target is represented by an arrow with the circular base corresponding to the mRNA and the arrowhead corresponding to the protein. The length of the arrow relates to the difference between mRNA and protein expression across the dataset.

### miRNA target prediction

Prediction of miRNA and oppositely correlated protein/mRNA interactions was performed using TargetScan 6.1 (http://www.targetscan.org/vert_61/) [14]. Only conserved targets were utilised and each predicted target assigned a rank according to the TargetScan algorithm quality measure known as the total context+ score.

### GO analysis

GO biological process enrichment analysis was carried out for the DE protein and mRNA lists via the DAVID interface (david.abcc.ncifcrf.gov).

## Abbreviations

miRNA: microRNA; CHO: Chinese hamster ovary; LC-MS/MS: Liquid chromatography mass spectrometry; mRNA: Messenger RNA; GO: Gene ontology; DE: Differentially expressed; RNA: Ribonucleic acid; UTR: Untranslated region; PCC: Pearson correlation coefficient; RBP: RNA binding protein; CIA: Coinertia analysis; Mab: Monoclonal antibody; GEO: gene expression omnibus; BH: Benjamini-Hochberg; ANOVA: Analysis of variance.

## Competing interests

The authors declare that they have no competing interests.

## Authors' contributions

NB, PM, MC, CC, MH, PD, ML and LZ contributed to the design of the research. PKI and LB cultured the cells. PM, MH, and SK conducted the proteomics expression profiling and proteomics differential expression analysis. PD and SA performed the microarray expression profiling. NB, NS and PKE carried out the miRNA expression profiling. CC and SFM carried out the CIA. CC conducted the miRNA and mRNA differential expression analysis, integrated the 3 data streams and drafted the manuscript. All authors read and approved the final manuscript.

## Supplementary Material

Additional file 1 **Sample Information. **Growth rates and " fast", "slow" designations for samples subjected to miRNA, mRNA and proteomic profiling. Click here for file

Additional file 2 **MicroRNA differential expression.** DE miRNAs identified following TLDA analysis. Also included are the PCCs utilised to identify 51 high priority miRNAs. Click here for file

Additional file 3 **Protein differential expression.** DE proteins identified using LC-MS/MS analysis and the BB-CHO proteomic database. Click here for file

Additional file 4 **mRNA differential expression.** DE mRNA identified using the CHO specific WyeHamster3a Affymetrix microarray. Click here for file

Additional file 5 **GO enrichment analysis.** Enrichment analysis of DE protein and mRNA lists against GO biological processes using DAVID. Click here for file

Additional file 6 ***in*-*silico *miRNA target prediction of Group A targets.** Identification of potential targets using TargetScan for candidate Group A. Click here for file

Additional file 7 ***in*-*silico *miRNA target prediction of Group B targets.** Identification of potential targets using TargetScan for candidate Group B. Click here for file
